# Supplementary material for: The Impact of Physical Activity Restrictions on Health-Related Fitness in Children with Congenital Heart Disease
Source: Int J Environ Res Public Health. 2022 Apr 7;19(8):4426. doi: 10.3390/ijerph19084426 (PMC9028029; doi:10.3390/ijerph19084426)
Supplement: Supplementary file 1 [file ijerph-19-04426-s001.zip › ijerph-1619582-supplementary.pdf]

Table S1. BMI-for-age Z scores differences according to PA restctions in boys and girls

PA restrictions	BMI Z scores (WHO)		BMI Z scores (CDC)		BMI Z score (WHO)		BMI Z score (CDC)	
	M ± SD	*p*	M ± SD	*p*	M ± SD	*p*	M ± SD	*p*
Physicians	**Boys**				**Girls**			
Any								
Restriction	−0.39 ± 1.03		0.54 ± 1.13		0.079 ± 1.075		−0.39 ± 1.03	
W/O restriction	0.074 ± 0.818	0.22	0.52 ± 1.27	0.21	0.14 ± 0.97	0.95	0.074 ± 0.818	0.86
Exertion								
Restriction	−0.38 ± 1.14		0.32 ± 1.30		−0.023 ± 1.16		−0.38 ± 1.14	
W/O restriction	0.19 ± 0.95	0.65	0.57 ± 1.16	0.39	0.15 ± 0.97	0.62	0.19 ± 0.95	0.65
Competition								
Restriction	−0.49 ± 1.11		0.50 ± 1.20		0.10 ± 1.02		−0.49 ± 1.11	
W/O restriction	0.0047 ± 0.79	0.19	0.54 ± 1.18	0.18	0.12 ± 1.00	0.91	0.0047 ± 0.79	0.97
Body contact								
Restriction	−0.48 ± 1.20		0.64 ± 0.91		0.26 ± 0.77		−0.48 ± 1.20	
W/O restriction	−0.022 ± 0.68	0.20	0.46 ± 1.33	0.18	0.026 ± 1.12	0.61	−0.022 ± 0.68	0.44
Parents								
Any								
Restriction	−0.24 ± 1.04		0.37 ± 1.31		−0.048 ± 1.21		−0.24 ± 1.04	
W/O restriction	0.16 ± 1.21	0.15	0.077 ± 1.46	0.20	−0.28 ± 1.34	0.29	0.16 ± 1.21	0.33
Exertion								
Restriction	0.11 ± 1.30		0.21 ± 0.97		−0.16 ± 0.92		0.11 ± 1.30	
W/O restriction	0.023 ± 1.15	0.84	0.20 ± 1.43	0.81	−0.19 ± 1.31	0.99	0.023 ± 1.15	0.96
Competition								
Restriction	−0.012 ± 1.01		0.35 ± 0.68		0.15 ± 0.67		−0.012 ± 1.01	
W/O restriction	0.044 ± 1.16	0.42	0.19 ± 1.44	0.463	−0.21 ± 1.32	0.78	0.044 ± 1.16	0.45
Body contact								
Restriction	−0.45 ± 1.19		0.37 ± 1.45		−0.051 ± 1.36		−0.45 ± 1.19	
W/O restriction	0.12 ± 0.92	0.07	0.13 ± 1.38	0.09	−0.24 ± 1.26	0.42	0.12 ± 0.92	0.48
Both								
Any								
Restriction	−0.27 ± 1.09		0.67 ± 1.33		0.23 ± 1.12		−0.27 ± 1.09	
W/O restriction	−0.11 ± 0.83	0.71	0.15 ± 0.67	0.62	−0.20 ± 0.60	0.23	−0.11 ± 0.83	0.24
Exertion								
Restriction	−0.053 ± 1.13		0.12 ± 1.09		−0.24 ± 1.02		−0.053 ± 1.13	
W/O restriction	−0.32 ± 0.92	0.54	0.62 ± 1.22	0.66	0.19 ± 1.01	0.33	−0.32 ± 0.92	0.32
Competition								
Restriction	−0.43 ± 1.20		0.45 ± 1.23		0.063 ± 1.04		−0.43 ± 1.20	
W/O restriction	−0.048 ± 0.80	0.37	0.58 ± 1.19	0.37	0.15 ± 1.02	0.75	−0.048 ± 0.80	0.80
Body contact								
Restriction	0.10 ± 1.17		0.83 ± 1.36		0.37 ± 1.18		0.10 ± 1.17	
W/O restriction	−0.33 ± 0.93	0.38	0.24 ± 0.96	0.50	−0.13 ± 0.79	0.12	−0.33 ± 0.93	0.12

Table S2. Physical competence according to PA restrictions in boys

PA restrictions	Locomotive Pctl		Object Pctl		TGMD-2 Pctl		Hand grip Z scores		S&R Z scores	
	M ± SD	*p*	M ± SD	*p*	M ± SD	*p*	M ± SD	*p*	M ± SD	*p*
Physicians										
Any										
Restriction	55.6 ± 30.5		67.2 ± 27.9		58.6 ± 29.9		−0.20 ± 0.83		2.34 ± 1.78	
W/O restriction	66.8 ± 28.8	0.34	52.2 ± 28.3	0.20	59.0 ± 29.1	0.97	0.35 ± 1.03	0.11	1.90 ± 2.14	0.55
Exertion										
Restriction	47.8 ± 31.5		56.7 ± 30.9		54.0 ± 31.8		−0.09 ± 0.86		3.23 ± 1.05	
W/O restriction	63.9 ± 28.7	0.18	65.1 ± 27.6	0.47	60.9 ± 28.3	0.57	0.01 ± 0.97	0.76	1.76 ± 1.98	0.04*
Competition										
Restriction	57.9 ± 31.6		54.5 ± 28.0		53.5 ± 27.0		−0.21 ± 0.89		1.90 ± 1.80	
W/O restriction	61.5 ± 29.9	0.77	79.2 ± 64.5	0.03*	68.3 ± 31.1	0.24	0.19 ± 1.09	0.32	1.67 ± 1.96	0.76
Body contact										
Restriction	62.7 ± 25.5		71.3 ± 28.3		63.7 ± 27.2		−0.04 ± 0.96		2.06 ± 1.79	
W/O restriction	56.3 ± 33.7	0.56	55.8 ± 27.5	0.16	55.0 ± 30.8	0.45	0.002 ± 0.92	0.89	2.32 ± 1.98	0.70
Parents										
Any										
Restriction	60.4 ± 27.3		64.2 ± 28.1		62.2 ± 30.2		−0.09 ± 0.87		1.81 ± 2.11	
W/O restriction	59.1 ± 27.6	0.84	60.5 ± 31.4	0.61	61.8 ± 26.9	0.95	0.15 ± 0.94	0.25	2.65 ± 1.46	0.03*
Exertion										
Restriction	63.3 ± 25.7		66.0 ± 29.2		67.6 ± 26.9		0.09 ± 1.24		1.47 ± 2.47	
W/O restriction	59.0 ± 27.7	0.64	61.1 ± 30.6	0.65	61.4 ± 29.4	0.55	−0.04 ± 0.86	0.67	2.52 ± 1.58	0.08
Competition										
Restriction	-		-		-		-		-	
W/O restriction	-	-	-	-	-	-	-	-	-	-
Body contact										
Restriction	59.7 ± 29.3		60.6 ± 30.9		62.4 ± 29.6		−0.01 ± 0.90		1.99 ± 1.95	
W/O restriction	59.5 ± 27.2	0.98	67.5 ± 28.9	0.47	60.2 ± 26.8	0.81	−0.09 ± 0.88	0.77	2.49 ± 1.65	0.32
Both										
Any										
Restriction	65.7 ± 29.0		42.8 ± 28.0		45.4 ± 24.7		0.52 ± 0.62		1.83 ± 1.73	
W/O restriction	63.9 ± 27.2	0.88	77.5 ± 22.1	0.01*	72.4 ± 29.4	0.04*	−0.03 ± 1.09	0.14	1.38 ± 1.96	0.58
Exertion										
Restriction	61.2 ± 31.8		59.8 ± 29.6		58.0 ± 26.7		−0.02 ± 1.32		1.71 ± 1.84	
W/O restriction	67.6 ± 25.9	0.61	73.6 ± 27.8	0.31	70.4 ± 35.4	0.38	0.28 ± 0.71	0.49	1.64 ± 1.82	0.94
Competition										
Restriction	64.5 ± 30.5		51.8 ± 27.7		54.5 ± 28.1		−0.21 ± 0.98		1.63 ± 1.85	
W/O restriction	65.5 ± 26.8	0.93	81.9 ± 21.9	0.01*	73.2 ± 49.3	0.18	0.44 ± 0.91	0.12	1.70 ± 1.81	0.93
Body contact										
Restriction	68.9 ± 26.3		57.8 ± 29.6		58.3 ± 32.1		0.41 ± 1.04		2.26 ± 1.37	
W/O restriction	63.8 ± 29.0	0.71	88.0 ± 18.6	0.002*	76.8 ± 20.3	0.16	0.07 ± 0.97	0.48	1.44 ± 1.91	0.28

***Significant difference (p<0.05)**

Table S3. Physical competence according to PA restrictions in girls

PA restrictions	Locomotive Pctl		Object Pctl		TGMD-2 Pctl		Hand grip Z scores		S&R Z scores	
	**M ± SD**	***p***	**M ± SD**	***p***	**M ± SD**	***p***	**M ± SD**	***p***	**M ± SD**	***p***
Physicians										
Any										
Restriction	44.2 ± 25.9		37.4 ± 29.9		33.4 ± 28.8		−0.29 ± 1.09		−0.84 ± 1.05	
W/O restriction	44.0 ± 27.6	0.98	50.5 ± 26.5	0.17	46.3 ± 27.8	0.17	0.13 ± 0.75	0.13	−1.28 ± 1.14	0.19
Exertion										
Restriction	45.0 ± 19.5		42.7 ± 28.1		36.7 ± 28.5		−0.56 ± 1.32		−0.79 ± 1.08	
W/O restriction	44.0 ± 27.5	0.93	41.9 ± 29.7	0.95	38.3 ± 29.3	0.90	−0.04 ± 0.90	0.16	−1.04 ± 1.10	0.53
Competition										
Restriction	48.3 ± 26.0		29.8 ± 28.0		30.3 ± 30.4		−0.27 ± 0.97		−0.86 ± 1.00	
W/O restriction	41.7 ± 28.0	0.45	52.4 ± 27.0	0.01*	44.6 ± 27.4	0.14	0.004 ± 0.87	0.35	−1.24 ± 1.10	0.27
Body contact										
Restriction	44.0 ± 26.4		41.0 ± 32.9		34.3 ± 30.3		−0.09 ± 1.05		−0.99 ± 1.18	
W/O restriction	44.2 ± 26.5	0.98	42.7 ± 27.2	0.85	40.3 ± 28.3	0.52	−0.22 ± 0.93	0.69	−1.00 ± 0.94	0.97
Parents										
Any										
Restriction	43.2 ± 30.8		32.8 ± 26.7		34.6 ± 29.7		−0.33 ± 1.05		−1.00 ± 1.06	
W/O restriction	51.4 ± 22.4	0.09	50.8 ± 24.4	0.0003**	50.5 ± 23.5	0.002**	0.19 ± 1.13	0.009*	−0.50 ± 1.19	0.01*
Exertion										
Restriction	27.3 ± 18.1		33.1 ± 30.6		24.6 ± 27.6		−0.57 ± 0.85		−0.71 ± 1.12	
W/O restriction	49.5 ± 26.1	0.02*	44.6 ± 26.4	0.27	45.7 ± 26.7	0.04*	0.02 ± 1.13	0.09	−0.70 ± 1.17	0.98
Competition										
Restriction	26.0 ± 27.0		13.1 ± 13.7		16.7 ± 21.8		−0.83 ± 0.76		−1.13 ± 1.16	
W/O restriction	49.6 ± 25.6	0.02*	45.9 ± 26.1	0.0003**	46.2 ± 26.5	0.005*	0.05 ± 1.13	0.008*	−0.67 ± 1.16	0.25
Body contact										
Restriction	46.3 ± 30.3		35.1 ± 25.1		37.5 ± 28.8		−0.17 ± 1.11		−0.87 ± 0.97	
W/O restriction	48.9 ± 24.8	0.65	46.8 ± 26.6	0.03*	46.7 ± 26.3	0.11	0.04 ± 1.13	0.32	−0.64 ± 1.22	0.33
Both										
Any										
Restriction	36.6 ± 26.6		37.3 ± 28.7		35.6 ± 29.8		−0.14 ± 0.89		−1.07 ± 1.00	
W/O restriction	47.9 ± 26.8	0.24	52.4 ± 29.7	0.15	43.2 ± 28.9	0.48	−0.15 ± 0.88	0.99	−1.36 ± 1.09	0.43
Exertion										
Restriction	37.6 ± 25.2		43.7 ± 28.9		38.5 ± 29.4		−0.38 ± 0.73		−1.20 ± 0.98	
W/O restriction	46.3 ± 27.4	0.44	41.2 ± 30.0	0.84	34.6 ± 30.9	0.75	−0.09 ± 0.91	0.44	−1.14 ± 1.05	0.88
Competition										
Restriction	48.7 ± 26.0		30.9 ± 28.2		30.4 ± 31.0		−0.20 ± 1.00		−0.93 ± 1.02	
W/O restriction	41.7 ± 27.8	0.43	50.4 ± 28.1	0.04*	43.8 ± 27.3	0.17	−0.10 ± 0.80	0.73	−1.33 ± 1.02	0.24
Body contact										
Restriction	48.2 ± 28.1		39.5 ± 29.9		35.5 ± 29.4		−0.22 ± 0.84		−1.24 ± 0.84	
W/O restriction	41.5 ± 26.0	0.44	44.1 ± 29.6	0.64	40.4 ± 29.9	0.61	−0.07 ± 0.92	0.62	−1.06 ± 1.19	0.59

***Significant difference (p<0.05)**

**** Significant difference after correction for multiple comparison (p<0.01)**
